# Editorial: Insights in health economics: 2021

**DOI:** 10.3389/fpubh.2022.966741

**Published:** 2022-07-26

**Authors:** Mihajlo Jakovljevic, Seiritsu Ogura

**Affiliations:** ^1^Institute of Advanced Manufacturing Technologies, Peter the Great St. Petersburg Polytechnic University, St. Petersburg, Russia; ^2^Institute of Comparative Economic Studies, Hosei University, Tokyo, Japan; ^3^Department of Global Health Economics and Policy, University of Kragujevac, Kragujevac, Serbia; ^4^Faculty of Economics, Hosei University, Tokyo, Japan

**Keywords:** Health Economics, health financing, health systems, emerging markets, BRICS, health expenditure, global south, sustainability

In the third decade of the twenty first century, methodological applications are leading to the movement of the knowledge frontier in the field of Health Economics. This frontiers Research Topic aims to highlight the latest advancements in health economics at the forefront of science. This editorial initiative, of particular relevance, led by Specialty Chief Editors of the Health Economics section Prof. Mihajlo Jakovljevic and Prof. Seiritsu Ogura, was focused on new insights, novel developments, current challenges, latest discoveries, recent advances, and future perspectives in the field of Health Economics (1).

The Topic has succeeded to invite a total of 18 forward-looking contributions. Some of them were describing the state of the art, outlining recent developments and major accomplishments that have been achieved. Also, possible directions of further development were designated in some of these articles. An array of contributing institutions were encouraged to identify the greatest challenges in the inter-disciplinary developments at the cross-sections of health economics, policy, global, and public health, pandemic health emergencies, quality of life, and mainstream market economics.

There was a set of mutually interconnected European contributions. An Eastern European study focused on Health Technology Assessment framework in this vast region (Daubner-Bendes et al.). Clench-Aas and Holte elaborated on the crisis of political trust in Europe and the relationship between income and life satisfaction. This problem was observed on a scale of different levels including the national, community, and individual citizen level. On the other hand, a Scottish national study worked on an Analysis of multiple risky health behaviors and associated disease outcomes using Scottish linked hospitalization data (Olajide et al.).

A pandemic-focused trial has revealed bottle neck inefficiencies of overstretched health systems. A convenient example was the study pointing out the relationship to real gross domestic product per capita in 38 European countries during the first wave of the pandemic (Pardhan and Drydakis). Another piece of complementary evidence coming from Japan has shown that the pandemic has severely impacted physician visits in Japan (Kumagai). One more Japanese piece of work has dealt with financial literacy, financial education, and smoking behavior (Watanapongvanich et al.). A single study of US origin focused on clustering and healthcare costs amongst the patients with multiple chronic conditions (Hajat et al.).

Chinese Universities encircle the largest set of studies that were successfully published in this Topic. Tang et al. have presented evidence of apparently puzzling connections between overweight/obesity and income-related inequality using Chinese labor force data. In another case a group led by Hao et al. has provided statistical evidence of the positive effect of the attempts of increased decentralization on the health outcome of communities, using DID analysis. Similarly, Xu and Lin published an elaborate econometric analysis of the effect of fiscal decentralization on the public health expenditure and public health, using continuous measures of fiscal decentralization. The fourth study is an interesting event-analysis of the food-industry firms that faced “crisis” from safety incidents, which examines the influence of their political connections and charitable donations, as well as the spill-over effects on their competitor firms (Xiang et al.). Particularly significant in an administrative system where health services almost never cross community boundaries is the study of spatial effect analysis of health spending patterns and trends in relationship to the health outputs in Chinese communities, establishing spill-over effects (Xu et al.). Finally, signaling the start of the expected and inescapable explosion of the demand for long-term care in the rapidly aging China, Tang et al. have provided a glimpse of the impact of long-term care insurance on the medical expenses and health status in Chinese society.

Vietnamese researchers did work on an interesting piece attempting to quantify the effects of various levels of dietary salt reduction on the prevalence of hypertension and the avoidable burden of stroke in Vietnam. These dynamic and complex consequences of salt intake in nutrition were observed through micro-simulation providing reliable assessment of the health and economic impacts (Aminde et al.). Another valuable Vietnamese contribution refers to a thorough exploration of complications of myocardial infarction after surgical treatment in Vietnam. This work contains an econometric part of the clinical equation, bringing valuable assessments of incremental cost, readmission risk, and duration of hospital admissions (Bui et al.). An Iranian study was particularly methodologically stringent adopting the framework of the systematic review and a meta-analysis. They managed to test the hypothesis of effectiveness of their national hospitals against the ongoing health sector evolution plan (Amini et al.).

Extensive Korean effort describes the market landscape and dynamics in relation to market exclusivity of the originator medicines in South Korea. This was conducted *via* adopting a retrospective cohort study design (Son). Last but not least, there was an ambitious piece of work originating from Sub-Saharan Africa. It was a comparative assessment of three national health systems namely the ones of Kenya, Tanzania, Uganda, and Zambia. This piece of research explores the causal relationship between donor commitments and disbursements for sexual and reproductive health aid (Kibira et al.).

The BRICS (Brazil, Russia, India, China, South Africa) Emerging markets continue to represent the engine of real economic growth worldwide. These nations shape the global demand for generic pharmaceuticals, medical goods, and services. Lock-downs caused by the pandemic have severely affected major supply chains and world trade routes. These changes alongside other upcoming challenges have decreased prospects for market recovery. The Global South nations, many of whom were represented in this topic, present a huge diversity in the historical legacy of their medical care financing and provision patterns. The burdens of premature mortality and absenteeism are multiplied by the prevalence and incidence of NCDs (2). Such health systems of LMICs countries will face difficult sustainability challenges due to long-term trends (3). An array of bottleneck vulnerabilities might be revealed once the entire health sectors are pushed to the limits of their resilience (4, 5). Current findings witness that the majority of global supply and demand is increasingly coming from the Asia—Pacific region. China, India, and South-East Asian ASEAN countries are most prominent representatives of this vast region (6).

Editors believe these valuable and diverse Topic contributions might open a new horizon of knowledge. Last but not least this is a unique opportunity to open the floor for a public debate on the Global South challenges from the perspective of academic health economics. The core aim of this special edition was to shed light on the progress made in the past decade in the Health Economics field (7). A diverse group of authors coming from academia, industry, governing authorities, and professional associations attempted to provide a thorough overview of the state of the art of the Health Economics field (8, 9). We hope that such an article Research Topic might inspire, inform, and provide direction and guidance to researchers in years to come.

## Author contributions

MJ has prepared the manuscript draft, while SO has revised it for important intellectual content. Both authors contributed to the article and approved the submitted version.

## Conflict of interest

The authors declare that the research was conducted in the absence of any commercial or financial relationships that could be construed as a potential conflict of interest.

## Publisher's note

All claims expressed in this article are solely those of the authors and do not necessarily represent those of their affiliated organizations, or those of the publisher, the editors and the reviewers. Any product that may be evaluated in this article, or claim that may be made by its manufacturer, is not guaranteed or endorsed by the publisher.
